# Complete genome sequence of esterase-producing bacterium *Croceicoccus marinus* E4A9^T^

**DOI:** 10.1186/s40793-017-0300-0

**Published:** 2017-12-21

**Authors:** Yue-Hong Wu, Hong Cheng, Ying-Yi Huo, Lin Xu, Qian Liu, Chun-Sheng Wang, Xue-Wei Xu

**Affiliations:** Key Laboratory of Marine Ecosystem and Biogeochemistry, Second Institute of Oceanography, State Oceanic Administration, 36th North BaoChu Road, Hangzhou, 310012 China

**Keywords:** *Croceicoccus marinus* E4A9^T^, Genome sequence, Esterase, *Alphaproteobacteria*

## Abstract

*Croceicoccus marinus *E4A9^T^was isolated from deep-sea sediment collected from the East Pacific polymetallic nodule area. The strain is able to produce esterase, which is widely used in the food, perfume, cosmetic, chemical, agricultural and pharmaceutical industries. Here we describe the characteristics of strain E4A9, including the genome sequence and annotation, presence of esterases, and metabolic pathways of the organism. The genome of strain E4A9^T^ comprises 4,109,188 bp, with one chromosome (3,001,363 bp) and two large circular plasmids (761,621 bp and 346,204 bp, respectively). Complete genome contains 3653 coding sequences, 48 tRNAs, two operons of 16S–23S-5S rRNA gene and three ncRNAs. Strain E4A9^T^ encodes 10 genes related to esterase, and three of the esterases (E3, E6 and E10) was successfully cloned and expressed in *Escherichia coli* Rosetta in a soluble form, revealing its potential application in biotechnological industry. Moreover, the genome provides clues of metabolic pathways of strain E4A9^T^, reflecting its adaptations to the ambient environment. The genome sequence of *C. marinus* E4A9^T^ now provides the fundamental information for future studies.

## Introduction

Lipolytic enzymes, including esterase (EC 3.1.1.1) and lipase (EC 3.1.1.3), are a general class of carboxylic ester hydrolases (EC 3.1.1), which catalyze the hydrolytic cleavage and formation of ester bonds [1, 2]. Esterase shows a preference for water-soluble short chain fatty acids (< 10 carbon atoms), while lipase prefers water-insoluble longer chain fatty acids (> 10 carbon atoms) [3, 4]. Many esterases do not require cofactors and have high stereospecificity toward chemicals, broad substrate specificity and high stability in organic solvents [4]. They are extensively used in the food, perfume, cosmetic, chemical, agricultural and pharmaceutical industries [5].

10.1601/nm.14628 [6], as a genus of the family 10.1601/nm.14015 [7], can be found in the marine environments, including deep-sea sediment, surface seawater and marine biofilm from a boat shell [6, 8, 9]. 10.1601/nm.14629 E4A9^T^, the type strain of the genus 10.1601/nm.14628, was isolated from deep-sea sediment collected from the East Pacific polymetallic nodule area [6]. The strain was able to produce esterase as well as lipase [6]. To get insight into the capability of esterase production, recently, we obtained the complete genome of 10.1601/nm.14629 E4A9^T^ and detected genes of esterase. This is the first genome report for the strain in the genus of 10.1601/nm.14628. We also describe the genomic sequencing related to its annotation for understanding their metabolic and ecological functions in the environment.

## Organism information

### Classification and features

10.1601/nm.14629 E4A9^T^ was isolated from a deep-sea sediment sample collected from the East Pacific polymetallic nodule area (8°22′38” N, 145°23′56” W) at a depth of 5280 m (temperature 2 °C, salinity 3.4%). Strain E4A9^T^ was obtained and routinely cultured on marine broth 2216 (MB, BD) at 30 °C. Subsequently polyphasic study of strain E4A9^T^ was performed. A new species 10.1601/nm.14629 gen. Nov. sp. nov. was proposed. Strain E4A9^T^ is the type strain of the species of 10.1601/nm.14629 [6], and was deposited into the China General Microbiological Culture Collection (10.1601/strainfinder?urlappend=%3Fid%3DCGMCC+1.6776 ^T^).

**Fig. 1:**
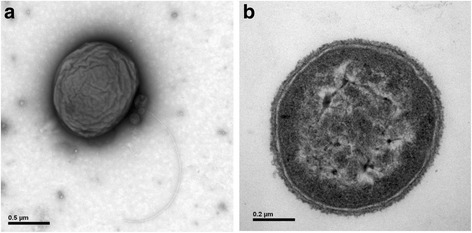
Transmission electron microscopy showing the cell morphology (**a**) and ultrastructure (**b**) of *Croceicoccus marinus* E4A9^T^. The flagella are present. Bars represent scales of 0.5 μm (**a**) and 0.2 μm (**b**), respectively

**Fig. 2:**
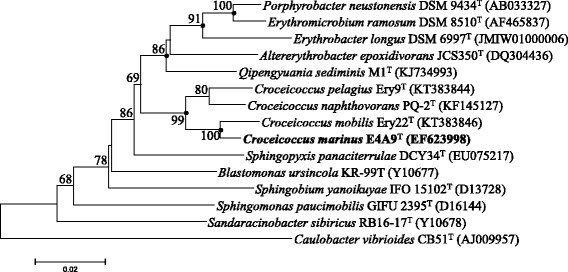
Phylogenetic tree based on 16S rRNA gene sequences was constructed by neighbor-joining algorithms. Related sequences were aligned with Clustal W. Evolutionary distances were calculated according to the algorithm of the Kimura two-parameter model. Bootstrap values (> 60%) based on 1000 replications are shown at branch nodes. Filled circles indicate that the corresponding nodes were also recovered in the trees generated with the maximum-likelihood and maximum-parsimony algorithms. Bar, 0.01 substitutions per nucleotide position

10.1601/nm.14629 [6] is a valid species belonging to the family 10.1601/nm.14015 [7], in the order 10.1601/nm.1164 [10, 11], class 10.1601/nm.809 [11, 12] and phylum 10.1601/nm.808 [13] *.* 10.1601/nm.14629 E4A9^T^ is a Gram-staining-negative and cocci-shaped bacterium (Fig. 1). It grew aerobically and used a series of organic carbon, such as _L_-arabinose, _D_-cellobiose, _D_-galactose and xylose, as sole sources of carbon and energy [6, 8]. Based on phylogenetic analysis of 16S rRNA gene sequence, the strain falls into the cluster comprising the 10.1601/nm.14628 species with a high bootstrap value (Fig. 2). Interestingly, strain E4A9^T^ could hydrolyze Tween 20, Tween 80 and tributyrin, indicating the presence of esterase as well as lipase [6]. The API ZYM system also supported the results that esterase (C4) and esterase lipase (C8) activities are present. The general features of strain E4A9^T^ was summarized in Table 1.

**Table 1:** Classification and general features of *Croceicoccus marinus* E4A9^T^ according to the MIGS recommendations [30]

MIGS ID	Property	Term	Evidence code^a^
	Classification	Domain *Bacteria*	TAS [31]
		Phylum *Proteobacteria*	TAS [12]
		Class *Alphaproteobacteria*	TAS [11]
		Order *Sphingomonadales*	TAS [10]
		Family *Erythrobacteraceae*	TAS [7]
		Genus *Croceicoccus*	TAS [6]
		Species *Croceicoccus marinus* (Type) strain: Strain E4A9^T^ (CGMCC 1.6776^T^= JCM 14846^T^)	TAS [6]
	Gram stain	Negative	TAS [6]
	Cell shape	Coccus	TAS [6]
	Motility	Motile	TAS [6]
	Sporulation	Non-sporulation	TAS [6]
	Temperature range	4–42 °C	TAS [6]
	Optimum temperature	28–30 °C	TAS [6]
	pH range; Optimum	6.0–9.0; 7.0	TAS [6]
	Carbon source	Organic carbon	TAS [6]
MIGS-6	Habitat	Deep-sea sediment	TAS [6]
MIGS-6.3	Salinity	Moderately halophilic, 0.5–10% NaCl	TAS [6]
MIGS-22	Oxygen requirement	Aerobic	TAS [6]
MIGS-15	Biotic relationship	Free-living	TAS [6]
MIGS-14	Pathogenicity	Non-pathogen	NAS
MIGS-4	Geographic location	East Pacific polymetallic nodule area	TAS [6]
MIGS-5	Sample collection	Not reported	
MIGS-4.1	Latitude	8°22′38” N	TAS [6]
MIGS-4.2	Longitude	145°23′56” W	TAS [6]
MIGS-4.4	Altitude	−5280 m	TAS [6]

^a^Evidence codes - *IDA* Inferred from Direct Assay, *TAS* Traceable Author Statement (i.e., a direct report exists in the literature), *NAS* Non-traceable Author Statement (i.e., not directly observed for the living, isolated sample, but based on a generally accepted property for the species, or anecdotal evidence). These evidence codes are from the Gene Ontology project [32]

## Genome sequencing information

### Genome project history

10.1601/nm.14629 E4A9^T^ [6] was selected for sequencing because it is relevant to genomic sequencing of the whole family of 10.1601/nm.14015 [7] and esterase production. The complete genome sequence was finished on May 29, 2015. The gap closure and annotation processes were performed by the authors. The GenBank accession number of the genome is CP019602, CP019603 and CP019604. The main genome sequence information is present in Table 2 and Table 3.

**Table 2:** Genome sequencing project information

MIGS ID	Property	Term
MIGS 31	Finishing quality	Finished
MIGS-28	Libraries used	10 kb
MIGS 29	Sequencing platforms	A PacBio RS II platform
MIGS 31.2	Fold coverage	248-fold
MIGS 30	Assemblers	HGAP Assembly version 2, Pacific Biosciences
MIGS 32	Gene calling method	GeneMarkS+ (NCBI)
	Locus Tag	A9D14
	Genbank ID	CP019602, CP019603, and CP019604
	GenBank Date of Release	June 13, 2017
	GOLD ID	Go0030822
	BIOPROJECT	PRJNA322659
MIGS 13	Source Material Identifier	CGMCC(China General Microbiological Culture Collection)
	Project relevance	Esterases production

**Table 3:** Summary of genome: one chromosome and two plasmids

Label	Size (Mb)	Topology	INSDC identifier	RefSeq ID
Chromosome	3.001363	Linear	CP019602.1	NZ_CP019602.1
Plasmid 1 (pCME4A9I)	0.761621	Linear	CP019603.1	NZ_CP019603.1
Plasmid 2 (pCME4A9II)	0.346204	Linear	CP019604.1	NZ_CP019604.1

### Growth conditions and genomic DNA preparation

10.1601/nm.14629 E4A9^T^ was aerobically cultivated in Marine Broth (MB, BD Difco™) at 30 °C and stored at −80 °C with 30% (*v*/v) glycerol. High-quality genomic DNA was extracted using the Qiagen DNA extraction kit, according to its protocol.

### Genome sequencing and assembly

The genome of strain E4A9^T^ was sequenced using SMRT technology with a PacBio RS II platform (Zhejiang Tianke Co. Ltd., China). One library was constructed with 10 kb insert size according to the large SMRTbell gDNA protocol (Pacific Biosciences, USA). The sequencing generated 85,372 reads with an average length of 11,938 nt (972 Mb, 248-fold genome coverage). The de novo assembly of the reads was performed using HGAP Assembly version 2 (Pacific Biosciences, USA). The circularization of final contigs was checked and the overlapping ends were trimmed.

### Genome annotation

The rRNA genes were found via RNAmmer 1.2 Server [14] and tRNA genes were identified using tRNAscan-SE 2.0 online server [15]. The open reading frames (ORFs) and the functional annotation of translated ORFs were performed using the RAST server online [16] and GeneMarkS+. Classification of some predicted genes were analyzed using COG database [17] and Pfam [18]. Genes with signal peptides were predicted using SignalIP 4.1 Server [19]. Genes with transmembrane helices were performed using TMHMM Server v. 2.0 [20]. The clustered regularly interspaced short palindromic repeats structures of the genomes were searched by CRISPRfiner program online [21]. Translated genes were assigned to Kyoto Encyclopedia of Genes and Genomes pathway using KEGG automatic annotation server with BBH method [22, 23]. The circular map of chromosome and plasmids were obtained using a CG View online server [24].

### Genome properties

**Fig. 3:**
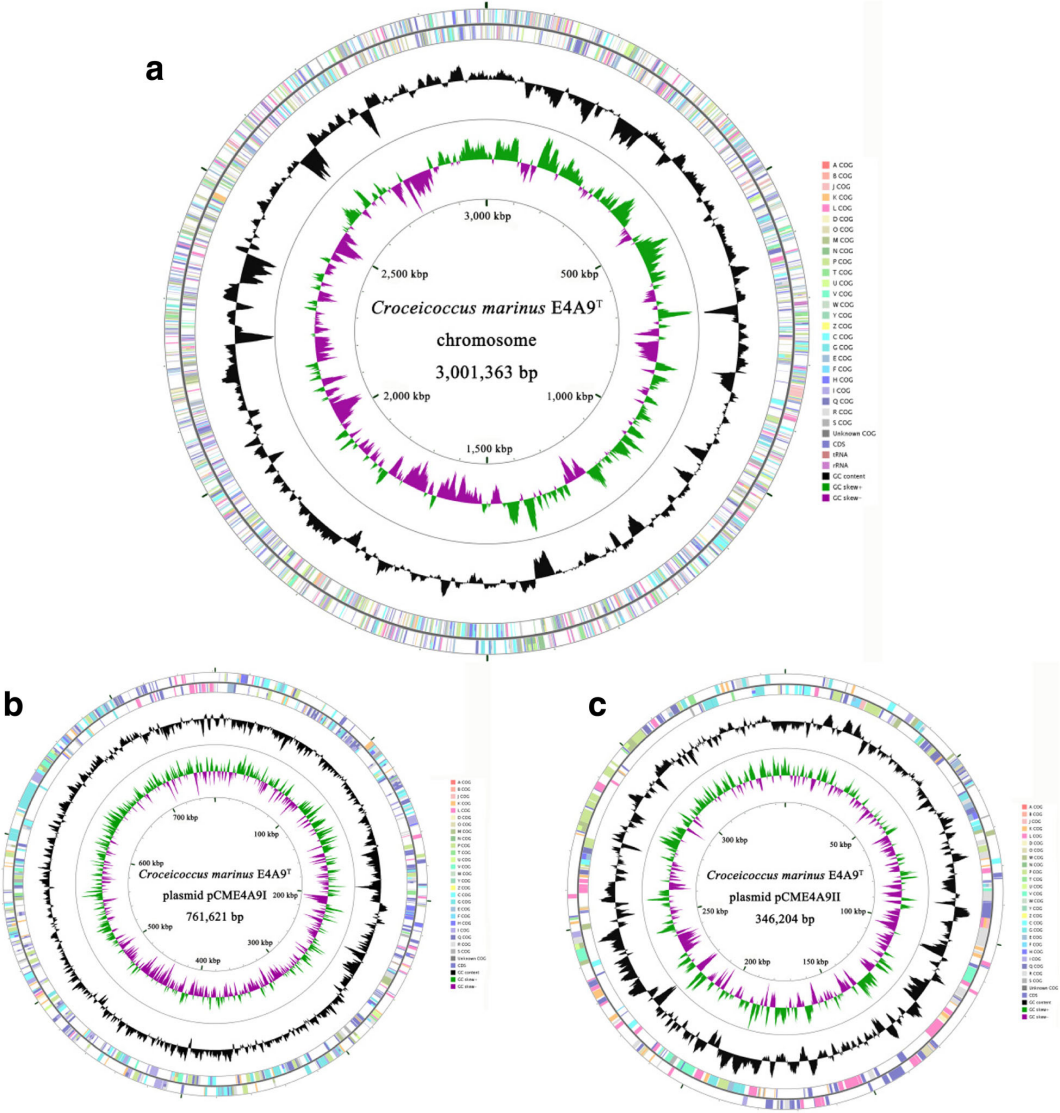
Circular map of the chromosome (**a**), plasmid pCME4A9I (**b**) and plasmid pCME4A9II (**c**). From outside to the center: CDSs and RNA genes on the forward strand (colored by COG categories), CDSs and RNA genes on the reverse strand (colored by COG categories), G + C content (peaks out/inside the circle indicate values higher or lower than the average G + C content, respectively), GC skew (calculated as (G-C)/(G + C), green/purple peaks out/inside the circle indicate values higher or lower than 1, respectively), genome size

The general features of strain E4A9 information are displayed in Table 1 and Table 2. The complete genome comprises 4,109,188 bp, with one chromosome (3,001,363 bp) and two large circular plasmids (plasmid pCME4A9I, 761,621 bp and plasmid pCME4A9II, 346,204 bp, respectively) (Fig. 3). The G + C content was 64.5 mol%. The genome of strain E4A9 contains 3653 coding sequences (CDSs), 48 tRNAs, two operons of 16S–23S-5S rRNA gene and three ncRNAs. Among the genes, 132 were assigned to pseudogene. The summary of features and statistics of the genome is shown in Table 4 and genes belonging to COG functional categories are listed in Table 5.

**Table 4:** Genome statistics of 10.1601/nm.14629 E4A9^T^

Attribute	Value	% of Total
Genome size (bp)	4,109,188	100
DNA coding (bp)	3,565,753	86.78
DNA G + C (bp)	2,650,881	64.51
DNA scaffolds	3	–
Total genes	3842	100
Protein coding genes	3653	95.08
RNA genes	57	1.48
Pseudo genes	132	3.47
Genes in internal clusters	517	13.46
Genes with function prediction	2699	70.25
Genes assigned to COGs	2827	73.58
Genes with Pfam domains	1566	40.76
Genes with signal peptides	304	7.91
Genes with transmembrane helices	755	19.65
CRISPR repeats	1	0.03

**Table 5:** Number of genes associated with general COG functional categories

Code	Value	%age^a^	Description
J	156	4.73	Translation, ribosomal structure and biogenesis
A	–	–	RNA processing and modification
K	190	5.76	Transcription
L	212	6.43	Replication, recombination and repair
B	1	0.03	Chromatin structure and dynamics
D	30	0.91	Cell cycle control, Cell division, chromosome partitioning
V	46	1.40	Defense mechanisms
T	168	5.10	Signal transduction mechanisms
M	193	5.86	Cell wall/membrane biogenesis
N	44	1.33	Cell motility
U	101	3.06	Intracellular trafficking and secretion
O	124	3.76	Posttranslational modification, protein turnover, chaperones
C	228	6.92	Energy production and conversion
G	187	5.67	Carbohydrate transport and metabolism
E	220	6.67	Amino acid transport and metabolism
F	64	1.94	Nucleotide transport and metabolism
H	146	4.43	Coenzyme transport and metabolism
I	199	6.04	Lipid transport and metabolism
P	174	5.28	Inorganic ion transport and metabolism
Q	111	3.37	Secondary metabolites biosynthesis, transport and catabolism
R	413	12.53	General function prediction only
S	289	8.77	Function unknown
–	770	23.36	Not in COGs

^a^The total is based on the total number of protein coding genes in the genome

Three replicons of the genome of strain E4A9, located in a circular chromosome and two large plasmids, were detected. Two plasmid replication initiator protein genes (ARU17925 and ARU18299) were found in the two plasmid sequence respectively, indicating that the genome of strain E4A9 contains two large circular plasmids. The G + C content of the two plasmids (63.5 mol% and 60.7 mol%, respectively) was a litter lower than that of the chromosome (65.2 mol%). The two plasmids have high gene density with 702 and 303 protein-coding regions, respectively. Many unsuspected genes involved in metabolism of aromatic compounds were identified in plasmid pCME4A9I. Almost 10% of the plasmid pCME4A9II sequence carries genes encoding gene of subsystem feature virulence, disease and defense, and most of them were of the copper homeostasis and cobalt-zinc-cadmium resistance. The functions of these genes are consistent with the notion that the two plasmids play an important role in the adaption of the bacteria in the sediment environment.

## Insights from the genome sequence

### Esterases presence of 10.1601/nm.14629 E4A9^T^

**Fig. 4:**
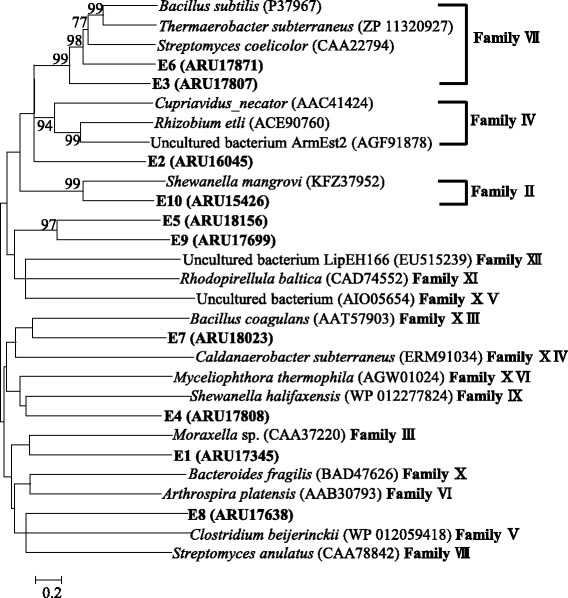
Maximum-likelihood phylogenetic tree based on esterases amino acid sequences. Bootstrap values (>60%) based on 1000 replications are shown at branch nodes

The presence of genes for the biotechnologically important enzymes like lipolytic enzymes were also predicted. Ten novel esterases were predicated (Fig. 4), and their amino acid sequences shared 58% to 85% identities to those of other lipolytic enzymes in the database. Phylogenetic analysis showed that predicated esterases E3 and E6 were grouped into family VII lipolytic enzymes and E10 was grouped into family II lipolytic enzymes. In order to investigate the biochemical properties of the esterases (E3, E6 and E10), recombinant plasmids were constructed and expressed in 10.1601/nm.3093 [25, 26]. After incubation of recombinant colonies for 48 h on the plate (Luria-Bertani agar medium) supplemented with 1% tributyrin, the three recombinant colonies had clear zones around the colonies. It indicated the presence of lipolytic activity. The calculated molecular weight of E3, E6 and E10 was 55.9, 46.1 and 22.4 kDa, respectively. The recombinant protein was soluble and purified using a Ni-NTA affinity chromatography column. The activity of purified E3, E6 and E10 was examined using *p*-nitrophenyl butyrate as substrate, and they had specific activities under standard reaction conditions (data not shown).

### Metabolism of 10.1601/nm.14629 E4A9^T^

The complete genome of 10.1601/nm.14629 E4A9^T^ was annotated for understanding the metabolic potentials based on the key genes of metabolic pathways of carbon, nitrogen, sulfur and phosphorus. (i) Carbon metabolism. The genome of strain E4A9^T^ is lack of carbon fixation and CO-oxidizing (*cox*) genes, indicating that the strain is not able to grow autotrophically. Strain E4A9^T^ can use organic carbon sources (Table 1). The genome has a complete glycolysis pathway (Embden-Meyerhoff-Parnas pathway). In addition, it possesses key genes of the Entener-Doudoroff pathway, the pentose phosphate pathway, and the tricarboxylic acid cycle. (ii) Nitrogen metabolism. The genome of 10.1601/nm.14629 E4A9^T^ possesses ammonium transporter genes and amino acids transporter genes (e.g. methionine and L-proline/glycine betaine). Genes encoding enzymes involved in polyamines biosynthesis are present, but the lack of polyamines transporters suggests its incapability of utilizing extracellular polyamines. Nitrate and nitrite transporters have been found in the genome of strain E4A9. It processes genes involved in nitrate and nitrite reduction (*nas*AB and *nir*BD, respectively) and is lack of genes involved in denitrification, nitrogen fixation and anammox. Thus, nitrate and nitrite could act as electron acceptors to generate ammonium, subsequently being utilized by strain E4A9 as a reduced nitrogen source. The genome of 10.1601/nm.14629 E4A9^T^ is lack of urease *(ure*ABC); however it harbors genes involved in urea decomposition, including urea carboxylase-related ABC transporter, urea carboxylase-related aminomethyltransferase, urea carboxylase and allophanate hydrolase, suggesting its capability of utilizing urea as a C or N source in the environment [27]. (iii) Sulfur metabolism. Strain E4A9^T^ possesses genes involved in assimilatory sulfate reduction (e.g. *cys*ND*, cys*C*, cys*H*, cys*JI). Sulfate can be reduced to sulfide, subsequently being incorporated into amino acids. Genes involving in alkanesulfonate assimilation (arylsulfatase and FMN reductase) are present in the genome of strain E4A9, suggesting its capability of utilizing organic sulfur compounds. However, it missed transporter genes for the uptake of extracellular alkanesulfonates. (iv) Phosphorus metabolism. Strain E4A9 is lack of genes for inorganic P storage as polyphosphate (*ppk*), as well as transport (*phnCDE*) and cleavage (*phnGHIJKLN*) of organic P in the form of phosphonates [28]. While strain E4A9 possesses the high-affinity phosphate transport system (*pst*SCAB) and regulatory genes (*pho*UBR), indicating an alternative strategy for maintaining a reliable supply of phosphorus [29].

## Conclusions

The complete genome sequence of 10.1601/nm.14629 E4A9^T^ contains a circular chromosome as well as two large circular plasmids and provides an insight into the genomic basis of its esterases production ability. Our data implies 10.1601/nm.14629 E4A9^T^ is a potential candidate in biotechnological application and facilitates the understanding for further industrial and biotechnological applications of esterases.

## Abbreviations

CDS: Coding sequence
CRISPRs: Clustered regularly interspaced short palindromic repeats
KAAS: KEGG automatic annotation server
KEGG: Kyoto encyclopedia of genes and genomes
ORF: Open reading frame
